# Supplementary material for: Asynchronous magnetic resonance elastography: Shear wave speed reconstruction using noise correlation of incoherent waves
Source: Magn Reson Med. 2022 Oct 27;89(3):990–1001. doi: 10.1002/mrm.29502 (PMC9792433; doi:10.1002/mrm.29502)
Supplement: Supplementary file 1 — DATA S1 MATLAB code used to generate the numerical simulations of Figure 1 [file MRM-89-990-s002.zip › k-Wave/helpfiles/example_pr_2D_adjoint.html]

Iterative Image Reconstruction Using The Adjoint Example (k-Wave)


# Iterative Image Reconstruction Using The Adjoint Example

This example gives a simple introduction to a very general and powerful class of techniques for image reconstruction. It uses gradient descent to minimise an error functional consisting of the difference between the data (the measured time series) and the output of a simulation. It is very similar to the Iterative Image Improvement Using Time Reversal Example.

- Open the file in the MATLAB Editor
- Run the file in MATLAB

## Contents

- Simulating the sensor data
- Running the adjoint model
- Updating the image
- Iterating the image reconstruction

## Simulating the sensor data

The example begins exactly as in the Iterative Image Improvement Using Time Reversal Example by defining an initial pressure distribution using a pre-defined image of the word 'k-Wave' and simulating sensor data from it using `kspaceFirstOrder2D`. Next, a set of modelled time series are initiated to zero:

```
% set the initial model times series to be zero
modelled_time_series = zeros(size(sensor_data));
```

## Running the adjoint model

The difference between the modelled and the measured signals are calculated. (At this stage this seems like a useless step as the modelled signals are zeros, but once this procedure begins to iterate, the modelled values will no longer be zero.) These difference time series are used as sources in the adjoint model, which is very similar to the time reversal approach except that the source is an `'additive'` type (the default in k-Wave), which adds the data to the existing field at each time point rather than replacing it. (The adjoint model itself is just the forward model with the source suitably defined.)

```
% set the source type to act as an adjoint source
source.p_mode = 'additive';
    
% calculate the difference between the measured and modelled data
difference = modelled_time_series - sensor_data;

% assign the difference time series as an adjoint source
% (see Appendix B in Arridge et al. Inverse Problems 32, 115012 (2016))
time_reversed_data = fliplr(difference);
source.p = [time_reversed_data(:, 1), time_reversed_data(:, 1), time_reversed_data(:, 1:end-1)] + ...
    [zeros(size(time_reversed_data(:, 1))), time_reversed_data(:, 1:end-1), 2 * time_reversed_data(:, end)];
	
% send difference through adjoint model
image_update = kspaceFirstOrder2D(kgrid, medium, source, sensor, input_args{:});
```

## Updating the image

The update to the image is simply a scaled version of the result from the adjoint calculation, where the choice of the scaling (the 'step length' in the gradient descent terminology) will determine the convergence of the iteration. Here it has just been fixed, but more sophisticated approaches to the minimisation would estimate a suitable value by calculating which value of the step length minimises the data error functional at each iteration. The image resulting from this first iteration is shown below.

```
% set the step length (this could be chosen by doing a line search)
nu = 0.25;

% update the image
p0_estimate = p0_estimate - nu * image_update.p_final;

% apply a positivity condition
p0_estimate = p0_estimate .* (p0_estimate >= 0);
```


## Iterating the image reconstruction

This image is then used in a forward model as a photoacoustic source term to generate a new set of modelled time series, and the procedure: finding the difference, running the adjoint, ... is repeated until the iteration has converged sufficiently. The image after five iterations is shown below.

```
% set the latest image to be the initial pressure in the forward model
source = rmfield(source, 'p');
source.p0 = p0_estimate;

% set the sensor to record time series (by default)
sensor = rmfield(sensor, 'record');

% calculate the time series at the sensors using the latest estimate of p0
modelled_time_series = kspaceFirstOrder2D(kgrid, medium, source, sensor, input_args{:});
```

Comparing this case to the Iterative Image Improvement Using Time Reversal Example suggests that the time reversal example converges faster, and indeed in this example it does. However, time reversal makes an assumption that the detectors form a continuous surface surrounding (or partially surrounding) the object. When that is not the case, for example when there are point detectors placed sparsely around the object, time reversal does not perform well. A data-matching approach, such as shown here, however, can still do very well.

Furthermore, additional terms can be added to the data functional that is being minimised here (so long as their gradients with respect to the unknown initial pressure values can be calculated), which makes for a far more general approach to the reconstruction. For example, prior knowledge of the sparsity of the image (in some domain) could be used to reduce the amount of data needed for a good image. Gradient descent is the simplest form of gradient-based minimisation and there is a large literature describing alternative methods for solving the image reconstruction problem using the adjoint model.
